# The relative contribution of drift and selection to phenotypic divergence: A test case using the horseshoe bats *Rhinolophus simulator* and *Rhinolophus swinnyi*

**DOI:** 10.1002/ece3.2966

**Published:** 2017-05-09

**Authors:** Gregory L. Mutumi, David S. Jacobs, Henning Winker

**Affiliations:** ^1^ Animal Evolution and Systematics Group (AES)Department of Biological SciencesUniversity of Cape TownCape TownSouth Africa; ^2^ Centre for Statistics in Ecology, Environment and Conservation (SEEC)Department of Statistical SciencesUniversity of Cape TownCape TownSouth Africa; ^3^ Department of BiologyUniversity of Massachusetts Amherst221 Morrill Science CenterAmherstMA01003USA; ^4^ South African National Biodiversity Institute (SANBI)Cape TownSouth Africa

**Keywords:** adaptation, diversification, Lande's model, micro‐evolutionary forces, natural selection, neutral evolution, speciation, vicariance

## Abstract

Natural selection and drift can act on populations individually, simultaneously or in tandem and our understanding of phenotypic divergence depends on our ability to recognize the contribution of each. According to the quantitative theory of evolution, if an organism has diversified through neutral evolutionary processes (mutation and drift), variation of phenotypic characteristics between different geographic localities (*B*) should be directly proportional to the variation within localities (*W*), that is, *B *∝* W*. Significant deviations from this null model imply that non‐neutral forces such as natural selection are acting on a phenotype. We investigated the relative contributions of drift and selection to intraspecific diversity using southern African horseshoe bats as a test case. We characterized phenotypic diversity across the distributional range of *Rhinolophus simulator* (*n = *101) and *Rhinolophus swinnyi* (*n =* 125) using several traits associated with flight and echolocation. Our results suggest that geographic variation in both species was predominantly caused by disruptive natural selection (*B* was not directly proportional to *W*). Evidence for correlated selection (co‐selection) among traits further confirmed that our results were not compatible with drift. Selection rather than drift is likely the predominant evolutionary process shaping intraspecific variation in traits that strongly impact fitness.

## 1 Introduction

Patterns of geographic phenotypic variation can reveal the relative contributions of different evolutionary processes on lineage diversification upon which biodiversity is based. If a species is distributed over a wide geographic area covering different habitats and biomes, populations in different geographic localities may be subjected to a variety of selection pressures and may experience varying degrees of isolation. Phenotypic divergence among localities may then ensue as a result of several processes acting on populations either separately, in combination or sequentially. For example, different populations may adapt to different local environmental conditions including differences in climate (e.g., rainfall and temperature), prey, and foraging habitat (Lomolino, Sax, Riddle, & Brown, 2006; Magurran, 1998; Morrone, 2009). Such divergence may be enhanced if gene flow is restricted by physical or biological barriers that may limit dispersal (Malhotra & Thorpe, 2000; Morrone, 2009). Alternatively, random events such as droughts, floods, and disease may decrease genetic variability in a population by decimating the population and leaving a few survivors carrying a subset of the original genetic variation (the bottle‐neck effect). Similarly, new populations established by a small number of individuals would also carry only a subset of the genome of the parent population (founder effect). Consequently, chance fixation of certain alleles is enhanced and other traits may be lost completely as a result of such genetic drift (Millstein, 2002; Wright, 1929). In both cases of adaptation and drift, if gene flow is restricted, divergence will be enhanced especially when founder populations are small. Even though phenotypic divergence may be driven by both natural selection and drift, most evolutionary explanations of divergence focus on adaptation (Weaver, Roseman, & Stringer, 2007). Studies which investigate the relative contributions of adaptation and drift are valuable because they provide a holistic understanding of how lineage divergence is initiated and proceeds in natural populations (Coyne & Orr, 2004; Orr & Smith, 1998).

Phenotypic divergence within species has been documented in several taxa, including animals that use acoustic signaling systems, such as insects, frogs, and mammals (Claridge & Morgan, 1993; Grant & Grant, 1989; Morton, 1977; Wilczynski & Ryan, 1999). Earlier explanations of such divergence were mostly based on natural selection (Schluter, 2009), whereas explanations based on drift, although already postulated in 1929 (Wright, 1929), have only relatively recently been put forward (Brandon, 2005; Millstein, 2002). However, there has been controversy on both the significance of drift to biological diversification and whether or not it can be distinguished from adaptation (Brandon, 2005; Brandon & Carson, 1996; Millstein, 2002).

Nevertheless, evidence for the role of drift has accumulated (Ackermann & Cheverud, 2002, 2004; de Azevedo, Quinto‐Sánchez, Paschetta, & González‐José, 2015; Lande, 1976; Smith, 2011; Weaver et al., 2007) and many studies have explored various methods to determine the relative contributions of adaptation and drift, for example, the rate test (Turelli, 1988), genetic approaches (Leinonen, O'Hara, Cano, & Merilä, 2008; Rogell, Eklund, Thörngren, Laurila, & Höglund, 2010; Sun et al., 2013), and quantitative genetic models (Ackermann & Cheverud, 2002, 2004; de Azevedo et al., 2015; Lande, 1976).

Phenotypic traits that perform crucial survival and reproductive functions form tight associations with environmental conditions, conferring fitness benefits on the bearers of such traits. This is especially so for sensory traits, which are highly sensitive to conditions within the environments through which sensory signals (e.g., acoustic signals) are propagated (Kirschel et al., 2011; Mutumi, Jacobs, & Winker, 2016; Sun et al., 2013). Echolocation is a sensory trait that is used not only in obstacle avoidance and prey capture (Schnitzler & Kalko, 2001) but also in mate choice (Puechmaille et al., 2014), and it is therefore likely to show signals for selection.

By necessity, adaptive complexes must exist between appendages used in maneuvering (e.g., wings) and sensory traits (e.g., acoustic signals) used to detect objects in the environment, if animals are to be able to avoid objects or to capture prey detected by their sensory systems (Norberg & Rayner, 1987). Such adaptive complexes exist between the wings and the acoustic signals of birds, for example, swiftlets and oil‐birds (Brinkløv, Fenton, & Ratcliffe, 2014; Fullard, Barclay, & Thomas, 1993; Griffin, 1958; Iwaniuk, Clayton, & Wylie, 2006; Konishi & Knudsen, 1979) and bats (Aldridge & Rautenbach, 1987; Jacobs, Barclay, & Walker, 2007) that echolocate. Because atmospheric attenuation is more pronounced at higher frequencies (Guillén, Juste, & Ibáñez, 2000; Mutumi et al., 2016) and given that bats use higher sound frequencies, associations within these adaptive complexes should be tighter in bats than in birds. These adaptive complexes can also include skull shape and size because it houses features for the production and reception of sensory signals while also functioning in handling and mastication of food. This has been evident in both mammals and birds (Freeman & Lemen, 2010; Jacobs, Bastian, & Bam, 2014). Nevertheless, several studies have also implicated drift in the evolution of acoustic signals that are used in reproduction rather than orientation, for example, in Neotropical singing mice (Campbell et al., 2010), anurans (Ohmer, Robertson, & Zamudio, 2009), and in birds (Irwin, Thimgan, & Irwin, 2008). Drift and selection may operate in tandem with their effects varying at different times and at different locations during the diversification of lineages (Orsini, Vanoverbeke, Swillen, Mergeay, & Meester, 2013).

The quantitative theory of genetic evolution as described by the Lande's model (Lande, 1976) has been applied to assess whether random evolutionary processes alone can explain phenotypic divergence (Ackermann & Cheverud, 2002, 2004; de Azevedo et al., 2015; Smith, 2011). The theory postulates a null model of drift, the rejection of which suggests that selection can be inferred (Smith, 2011). In the Lande's model, patterns of variance/covariance (within and between localities) of phenotypes are used to assess the contribution of drift. Accordingly, if an organism has diversified through neutral evolutionary processes (mutation and drift), variation of phenotypic characteristics between different geographic localities (*B*) should be directly proportional to the variation within localities (*W*), that is, *B *∝* W* (Ackermann & Cheverud, 2002). Significant deviations from such proportionality imply that non‐neutral forces (natural selection) are responsible for the divergence of populations. For example, using this approach, the roles of both drift and selection were identified in the skulls of primates (Marroig & Cheverud, 2004). Although strong selective forces were also identified in some regions of the human skull (de Azevedo et al., 2015), genetic drift was shown to be the primary process in the diversification of facial features and skull structure of the genus *Homo* (Ackermann & Cheverud, 2000; Smith, 2011) and in the skull morphology of monkeys (Marroig & Cheverud, 2004; Marroig, Vivo, & Cheverud, 2004). Thus, adaptive explanations may be over‐represented if not weighed against a null model of drift (Marroig & Cheverud, 2004). Surprisingly few studies have used this approach.

Bats offer an interesting test case for assessing the relative roles of drift and selection on nonprimate mammals using Lande's model. Bats exist in almost every known biome with the majority of species having wide distributional ranges covering several habitats and even spanning biomes (Csorba, Ujhelyi, & Thomas, 2003; Monadjem, Taylor, Cotterill, & Schoeman, 2010). Variations in habitat conditions likely impose an array of selective forces on a phenotype that is likely fine‐tuned to specific habitats owing to the intricacies of the adaptive complex; comprising adaptations for flight, echolocation, and feeding (Norberg & Rayner, 1987). This has been shown by adaptive trends in wing morphology which parallel those in echolocation call structure and skull morphology in several families of bats (Jones, 1999; Norberg & Rayner, 1987). Despite being volant, the dispersal ability of bats is limited and gene flow can be restricted (Moussy et al., 2013). Vicariance as a result of barriers in the form of water bodies, extensive human developments, and mountain ranges can therefore split bat populations into smaller ones. Drift may therefore play a role in the evolution of phenotypic traits in such small populations (Whitlock, 2000) even if those traits have fitness implications.

Horseshoe bats (Rhinolophidae) have wide geographic distributions across spatially heterogeneous environments in southern Africa (Csorba et al., 2003; Monadjem et al., 2010). Furthermore, they vary in population size from relatively small (tens of individuals) to relative large (thousands of individuals) as well as in body size, dispersal capabilities, and the degree to which they are philopatric (Kunz & Parsons, 2009). Geographic variation in the resting frequency of the echolocation calls of many horseshoe bats has been shown to be mainly the result of adaptations to optimize sound propagation in habitats of varying atmospheric conditions, for example, humidity and temperature (Bazley, 1976; Guillén et al., 2000; Huffman, 1992) and obstacles which have to be avoided during flight, for example, vegetation (Odendaal, Jacobs, & Bishop, 2014; Xu et al., 2008). It has been suggested that body size and wing dimensions covary with resting frequency as a consequence of optimization of flight and echolocation in habitats of varying clutter and prey (Jacobs et al., 2007; Norberg & Rayner, 1987).

We investigated the relative contributions of adaptation and drift in phenotypic divergence associated with flight and echolocation characteristics in two horseshoe bats, *Rhinolophus simulator* and *Rhinolophus swinnyi* that were similar in size but differed in call frequency (Mutumi et al., 2016) using Lande's model (Lande, 1976) adapted by Ackermann and Cheverud (2002) for phenotypic traits. We tested the hypothesis that selection rather than drift should be the predominant process in the evolution of traits associated with flight and sensory systems because, to be functional, these traits have to comply with the physical laws of aerodynamics and signal propagation. We evaluated the following predictions: (1) Lande's model would yield signals of selection through the rejection of the null model of drift for traits associated with flight and echolocation; (2) the relative importance of drift and selection will vary across the different traits and different geographic localities (de Azevedo et al., 2015); (3) the signal for selection will be stronger for *R. swinnyi* than for *R. simulator* because *R. swinnyi* uses higher call frequencies which are likely affected more by atmospheric attenuation.

## 2 Methods

### 2.1 Study sites and animals

Bats were sampled from caves and disused mine‐shafts across the distributional ranges of the two focal species *Rhinolophus simulator* (10 localities) and *Rhinolophus swinnyi* (nine localities) along a latitudinal gradient ranging from 16°S to 32°S (fig. 1 in Mutumi et al., 2016). *Rhinolophus simulator* and *R. swinnyi* use high duty cycle echolocation calls dominated by a constant frequency component at means of 80 and 107 kHz, respectively (see fig. [Link], [Link] in Mutumi et al., 2016). The two species occur across seven woodland types which allowed us to assess the effect of habitat variation. The study sites were located in the eastern half of southern Africa, ranging from Zambia in the north, through Zimbabwe and Botswana into South Africa in the south. The northernmost locality was the Central Zambezian Miombo woodland in Zambia. The central localities include the Zambezian and Mopane woodlands, Southern Miombo woodlands, and the Eastern Zimbabwe Montane Forest‐grassland Mosaic, in Zimbabwe. South African populations occur within Highveld grasslands. The Botswana populations occur within an ecotone of three woodlands: Kalahari Acacia‐Baekiaea, Kalahari Xeric Savannah, and Southern Africa Bushveld. Climates differ between woodlands with the site in Botswana being the driest and the Eastern Zimbabwe Montane Forest‐grassland Mosaic, the wettest (Olson et al., 2001). We used the same sampling methods as in Mutumi et al. (2016).

### 2.2 Morphology and echolocation measurement

Several body, wing, and head measurements (Table 1) were taken from captured live bats. These measurements were taken based on their ecological significance and the precision with which they could be measured on live bats in the field. Forearm length (FA), and other morphometric characters (Table A1) were measured to the nearest 0.1 mm using dial calipers and body mass (to the nearest 0.5 g) using a portable electronic balance. The right wing of each bat was extended on graph paper as per Saunders and Barclay (1992), and photographed using a digital camera (Canon Powershot A540, Canon inc, Malaysia) positioned at an angle of 90° and a distance of 30 cm above the wing and parallel to a flat table top. This minimized angular distortion so that length measurements and wing area could be measured using Sigma‐Scan Pro 5 version 3.20 (SPSS Inc., Cary, NC, USA). The graph paper was used to calibrate Sigma Scan. From the images, wing area was calculated as the area of the two wings, the tail membrane and the body between the wings, that is, excluding the head (Norberg & Rayner, 1987). Wingspan was taken as the distance between wingtips of fully extended wings (Norberg & Rayner, 1987). Wing loading was calculated in Newtons per square meter (N m^−2^) as the weight (mass in kg × acceleration due to gravity in m s^−1^) divided by the wing area (in m^2^) as in Norberg and Rayner (1987). Aspect ratio was calculated as the square of the wingspan divided by the wing area (Tables 1 and A1; Norberg & Rayner, 1987). Echolocation calls were recorded and analyzed as described in Mutumi et al. (2016).

**Table 1:** Phenotypic parameters measured from live bats in the field, *Rhinolophus simulator* and *R. swinnyi*

Abbreviation	Name	Description
RF	Resting frequency	Peak frequency of the constant frequency component of the call measured in kilohertz (kHz) from the power spectrum
FA	Forearm length	Forearm length measured in millimeters
TR	Upper tooth‐row length	Upper tooth‐row length (measured in millimeters) from the end of the last molar to the front‐end of the first molar
HH	Head height	Head‐height (measured in millimeters) from beneath the jaw just in front of the auditory bulla to the highest point of the head
HW	Head width	Maximum width of the head measured in millimeters across the head just behind the two ears
HL	Head length	Condylobasal length (measured in millimeters) from the tip of the nose tip to the skull lambda
FL	Foot length	Foot length measured (measured in millimeters) to the point of where the nail emerges
TL	Tail length	Distance from the tip of the tail to anus measured in millimeters
WS	Wingspan	Wing span length measured in millimeters between the tips of the outstretched wings including the body
WA	Wing area	Wing area measured in square meters as the combined area of the two wings, the tail membrane and the portion of the body between the wings
AR	Aspect ratio	Calculated as the square of the wingspan in meters divided by the wing area in square meters
WL	Wing loading	Calculated as the weight divided by the wing area and is measured in Newtons per square meter (N m^−2^)

### 2.3 Statistical methods

Data were first transformed using mean standardization to equalize the scale of our variables (Jacobs et al., 2013) in R statistics (R Development Core Team, 2013). Only parametric tests for subsequent analyses were used because the majority of our variables satisfied normality and homogeneity of variances among populations as in Ackermann and Cheverud (2002) and in Ackermann and Cheverud (2004). Furthermore, variance patterns rather than absolute sizes are central to the approach we used, so that minor violation of homogeneity of variances is generally not considered a major concern (de Azevedo et al., 2015).

### 2.4 Sexual dimorphism

Sexual dimorphism was assessed using ANOVA (Siemers, Beedholm, Dietz, Dietz, & Ivanova, 2005) with the phenotypic variables as multivariate response variables. Dependent variables, sex, and site were specified as categorical predictors. Univariate results for each variable were used to assess sexual dimorphism.

### 2.5 Geographic variation

To investigate the degree of geographic variation among samples from different localities, a discriminant function analysis (DFA) was performed with the phenotypic variables as dependants and populations as independent variables. To avoid multicollinearity of independent predictors, phenotypic variables were first converted into principal component scores (PCs) by means of principal component analysis (PCA). From the DFA using principal component scores (PCs) as input variables, squared Mahalanobis distances between populations were extracted in bivariate space from the first two functions. To illustrate how localities separated in two‐dimensional phenotypic space, multidimensional scaling plots were applied to the squared Mahalanobis matrix of phenotypic distances. Additionally, a cluster diagram was generated for each species to gauge how the localities grouped based on their phenotype differences. The phenotypic distance matrix was also regressed against the geographic distance matrix (calculated from geographic coordinates—straight‐line distances) to determine whether the geographic patterning was driven by isolation by distance using the Mantel test in R statistics (R Development Core Team, 2013), package Ade4 (Dray & Dufour, 2007).

### 2.6 Lande's model

Selection among localities of *R. simulator* and *R. swinnyi* was tested by attempting to reject the null model of drift which is based on Lande's model (Lande, 1976, 1979). To use phenotypic instead of genetic traits, for which this model was originally developed, the version developed by Ackermann and Cheverud (2002), known as the beta‐test, was employed. Accordingly, phenotypic within‐group covariance matrices (*P*) instead of genetic covariance matrices (*G*) were employed (Ackermann & Cheverud, 2002; de Azevedo et al., 2015). A known relationship has been established between the two kinds of matrices *P* and *G* (Cheverud, 1988; Roff, 1996), so that *P* can be used to approximate *G* (de Azevedo et al., 2015). The model specifies that if populations have diversified through neutral evolutionary processes (mutation and drift), variation of phenotypic characteristics between populations (*B*) should be directly proportional to the variation within populations (*W*), that is, *B* ∝ *W* (Ackermann & Cheverud, 2002). Significant deviations from this null model imply other non‐neutral forces acting on the phenotype of the species, possibly natural selection.

The process of deriving *B* and *W* was programmed in R and the code developed to implement Lande's model for phenotypic traits is provided in the supplementary material (Data S1) together with an example dataset for *R. simulator* (Data S2). First, *W* was estimated through MANOVA with 12 phenotypic traits (Tables 1 and A1) as the dependent variables. To analyze whether drift may play a role in diversifying morphology, but not frequency, we ran parallel analyses with and without RF to compare the results (also provided in the R script [Data S1]). We could not partition our analyses (further than the RF‐morphology partition) into different functional units (e.g., flight apparatus, head, and body appendages) because only a few variables per functional complex could be taken without compromising the health of the bats under difficult field conditions.

Population (locality) and sex were specified as the independent variables. From the MANOVA, the residual variance/covariance (V/CV) matrix was extracted (Ackermann & Cheverud, 2004). This matrix provides an estimate of the portion of variation that remains unexplained by interpopulation and sexual differences (some parameters were sexually dimorphic). A set of PCs from the V/CV matrix using PCA was generated and eigenvalues of the PCs were extracted to represent the within**‐**population variance *W*.

Next, *B* was estimated by multiplying the matrix of eigenvectors (obtained from the PCA on the V/CV matrix) by the matrix of the trait means for each locality (trait = columns; population = rows). The product of these two matrices yielded a “new” set of PCs. The variances associated with each new PC score were calculated as the mean square of scores within each PC. This variance value represents the between group variance *B* for each new PC. The natural logarithms of *B* were regressed against those of *W* to assess whether the between locality variance could be fully explained by the within locality variance, that is, whether the regression slope (*b*) was significantly different from a gradient of one.

The β‐test also predicts that the new PCs (calculated from eigenvectors and trait means, as described above) should remain uncorrelated if drift is present. Pearson's correlation test was therefore used as further confirmation of drift (Ackermann & Cheverud, 2002; de Azevedo et al., 2015). If the PCs are correlated, there is a possibility of coselection on the corresponding traits (de Azevedo et al., 2015).

### 2.7 Lande's model: stepwise exclusion of components

Some populations/PCs were sequentially excluded and the model reran after each exclusion to explore how each specific population or phenotype influenced the slope of the regression of *B* on *W*, because drift or selection may be differentially exerted on populations occurring in different habitats or across different phenotypes. This rationale was also based on similar reasoning as in de Azevedo et al. (2015) when they excluded a PC at a time to assess whether different regions of human skulls differentially experienced drift/selection. Lande's model was therefore repeated excluding (1) a population at a time, (2) a PC at a time, and (3) a combination of populations and PCs. When a single population was removed, the whole analysis procedure was repeated from the MANOVA stage up to the regression. Excluding a PC at a time was performed only at the regression stage, meaning that only this stage would be repeated without a particular PC. Therefore, all populations were used in the analysis up to the regression after which the regression was repeated several times excluding a PC at a time, to identify changes in the relationship between *B* and *W* with and without each trait in terms of the gradient of the regression between *B* and *W*. Some PCs were also removed from the analyses where we excluded a population at a time and the regression was rerun between *B* and *W*. In this case, only the last PC carrying the lowest eigenvalue was excluded to simplify the analyses. These analyses also tested the influence of sample size and outliers on our results.

## 3 Results

The morphology and resting frequency (RF) of 101 *R. simulator* and 125 *R. swinnyi* (Table A1) were analyzed.

### 3.1 Sexual dimorphism

MANOVA results showed that both sex and localities were phenotypically different within both species (*R. simulator*: MANONA sex *F*_12;73_ = 3.74; *p* < .001; Locality *F*_96;502_ = 7.21; *p* < .001. *R. swinnyi* Sex: *F*_12;100_ = 5.34; *p* < .001 and Locality *F*_84;620_ = 5.09; *p* < .001). Only four of the 12 variables in the two species were sexually dimorphic as indicated by significant effects based on a significance level of α = 0.05 (WS: ANOVA: *F*_1;93_ = 7.7; *p* < .001, WA: ANOVA: *F*_1;93_ = 6.9; *p* < .05, WL: ANOVA: *F*_1;93_ = 4.5; *p* < .05 and RF: ANOVA: *F*_1;93_ = 11.7, *p* < .001 for *R. simulator* and, WS: ANOVA: *F*_1;119_ = 6.7; *p *< .05, WA: ANOVA: *F*_1;119_ = 5.7; *p* < .05, and RF: ANOVA: *F*_1;119_ = 47.9; *p* < .001 for *R. swinnyi*). Both species exhibited dimorphism for the same parameters except in the case of WL which was only dimorphic in *R. simulator*. For Lande's model, sex was incorporated as a categorical predictor together with study sites, and variation due to sex differences was therefore taken out of the within‐population V/CV matrix used in the modeling. For the exploratory stages (DFA), we balanced the proportions of the sexes for populations which were dimorphic.

### 3.2 Geographic variation

**Figure 1:**
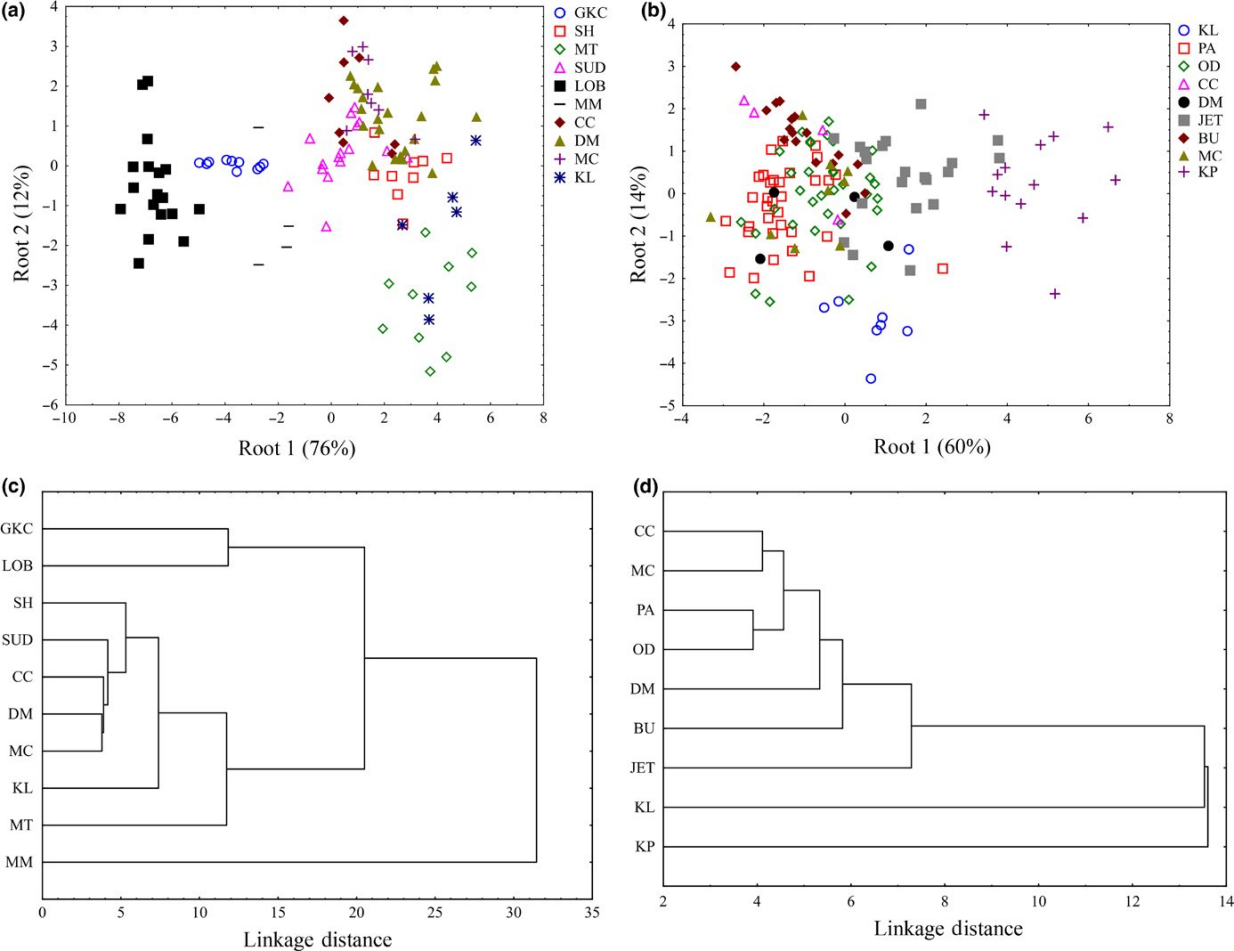
Multidimensional scaling (MDS) plots for (a) *Rhinolophus simulator* and (b) *Rhinolophus swinnyi* and cluster diagrams for (c) *Rhinolophus simulator* and (d) *Rhinolophus swinnyi* based on squared Mahalanobis distances showing interpopulation variation in phenotype (based on body size, flight morphology, and echolocation parameters). Localities: South Africa; PA = Pafuri, GKC = Gatkop Cave, SUD = Sudwala. Zimbabwe; CC = Chinhoyi, JET = Jiri Estate – Triangle, MT = Matopo, OD = Odzi German Shafts, DM = Dambanzara, MC = Mabura Cave, KP = Kapamukombe. Zambia; KL = Kalenda, SH = Shimabala, Mozambique; BU = Bunga Forest, MM = Monaci Mine, Botswana; LOB = Lobatse.

Geographic variation in phenotype was indicated for both species (Figure 1). There was a fairly distinct separation of study sites in the 2D phenotypic space (using canonical roots 1 and 2 from the DFA), with LOB, GKC, and MM the most distinct from the rest in *R. simulator* (Figure 1a), whereas KP was the most distinct site from the rest in *R. swinnyi* (Figure 1b). Total classification success reached 84.7% for *R. simulator* (Wilks’ Lambda: 0.0038, *F*_108;667_ = 7.1333; *p* < .001) and 80.4% for *R. swinnyi* (Wilks’ Lambda: 0.0324, *F*_96;838_ = 5.8034; *p* < .001). In *R. simulator*, canonical roots 1 and 2 accounted for 88% of the variation. Root 1 explained 76% of the variation and was predominantly made up of WS, WA, AR, and RF. Root 2 explained 12% of the variation and was predominantly made up of WS, WA, and AR. These three variables were all associated with flight and detection and suggest differences in maneuverability and orientation.

*Rhinolophus swinnyi*'s canonical roots 1 and 2 from the DFA accounted for 74% of the variance. Root 1 explained 60%, predominantly made up of FA, HL, TL, WL, and RF. Root 2 accounted for 14% and was predominantly made up of HL, WS, WA, WL, and RF. These are variables associated with body size, flight, and detection and suggest differences in size, maneuverability, and orientation.

Cluster diagrams showed a hierarchy in the phenotypic linkage distances as a measure of dissimilarity between sites. Following this hierarchy, the sites could be arranged in order of greatest dissimilarity to the rest as follows: *R. simulator*; MM, (GKC, LOB), MT, KL, SH, SUD, CC, DM, MC, and *R. swinnyi*; KP, KL, JET, BU, DM, (OD, PA), (MC, CC), (Figure 1c,1d, respectively).

According to the Mantel test results, geographic variation in phenotype among localities of the two species was not related to the geographic distances among them (*R. simulator*: Monte‐Carlo test, Observation: 0.362, Simulated *p* value: .075 and *R. swinnyi*, Monte Carlo test, Observation: −0.082, Simulated *p* value: .534: Based on 10,000 replicates).

### 3.3 Lande's model results

**Figure 2:**
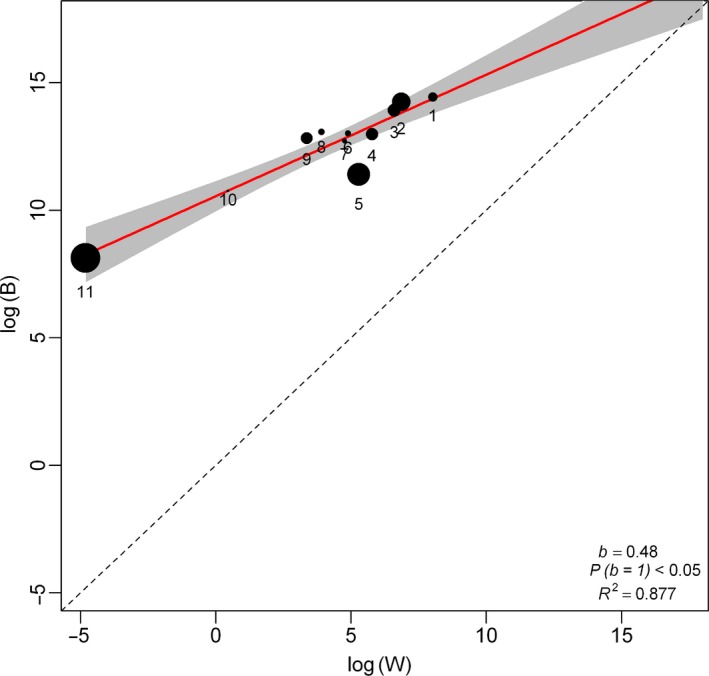
Regression of *B* (between‐group) and *W* (within‐group variance) for *Rhinolophus simulator*. PCs generated using all variables including resting frequency (RF; ref Table 1). Dot sizes indicate the PC's relative influence on the regression slope (calculated as the difference between the slope values with and without that particular PC point). The regression line (red line) is compared to the null hypothesis of drift *b* = 1 (dashed line)

**Figure 3:**
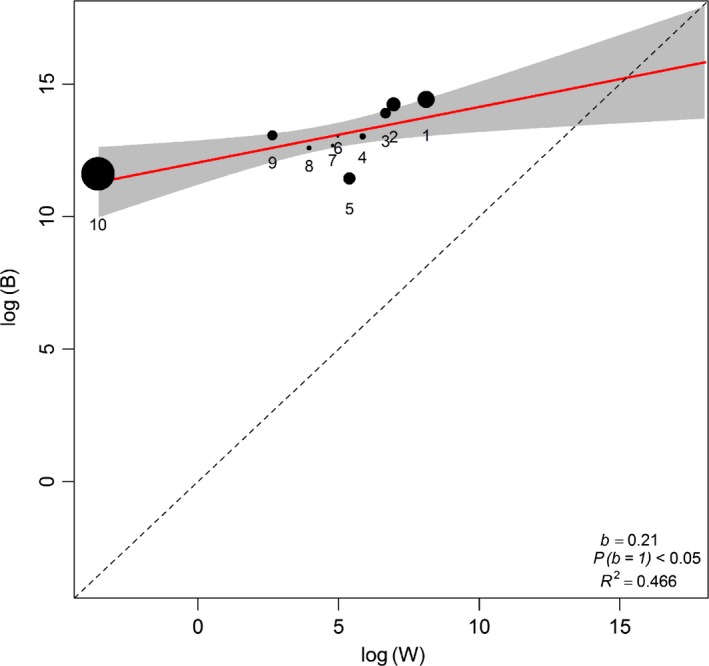
Regression of *B* (between‐group) and *W* (within‐group variance) for *Rhinolophus simulator*. PCs generated using all variables except resting frequency (RF; ref Table 1). Dot sizes indicate the PC's relative influence on the regression slope (calculated as the difference between the slope values with and without that particular PC point). The regression line (red line) is compared to the null hypothesis of drift *b* = 1 (dashed line)

**Figure 4:**
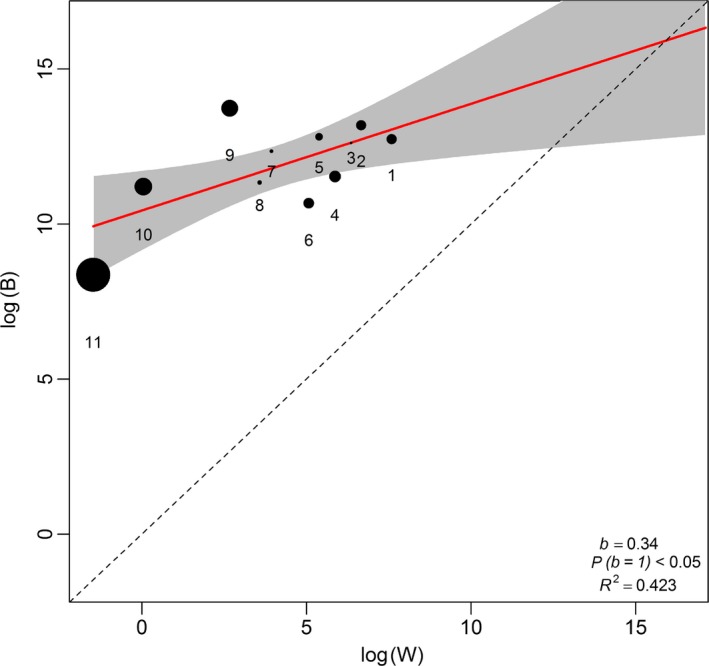
Regression of *B* (between‐group) and *W* (within‐group variance) for *Rhinolophus swinnyi*. PCs generated using all variables including resting frequency (RF; ref Table 1). Dot sizes indicate the PC's relative influence on the regression slope (calculated as the difference between the slope values with and without that particular PC point). The regression line (red line) is compared to the null hypothesis of drift *b* = 1 (dashed line)

**Figure 5:**
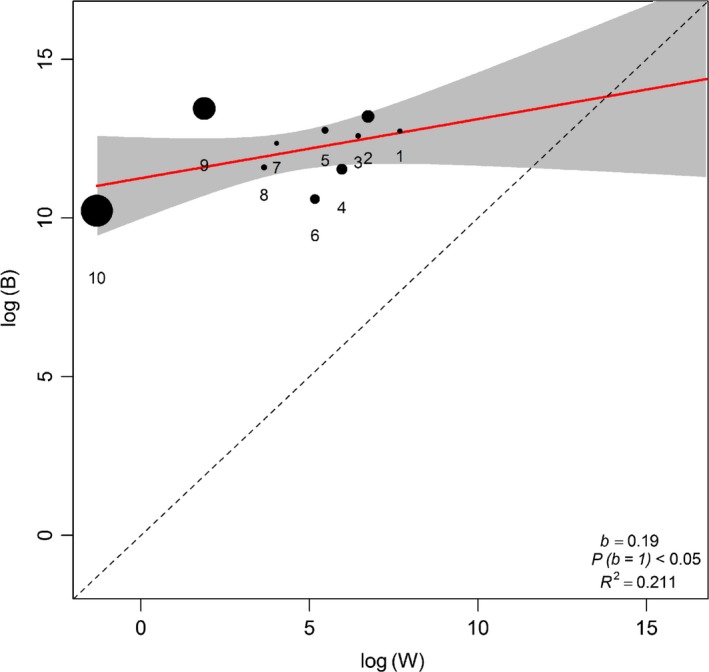
Regression of *B* (between‐group) and *W* (within‐group variance) for *Rhinolophus swinnyi*. PCs generated using all variables except resting frequency (RF; ref Table 1). Dot sizes indicate the PC's relative influence on the regression slope (calculated as the difference between the slope values with and without that particular PC point). The regression line (red line) is compared to the null hypothesis of drift *b* = 1 (dashed line)

Within‐locality variances could not explain between locality variances, because *B* was not directly proportional to *W* in all the tests (Tables 2 and 3; Figures 2, 3, 4, 5). This result did not change when RF was excluded from the analysis (compare Figures 2 and 3 for *R. simulator* and Figures 4 and 5 for *R. Swinnyi;* with and without RF, respectively). Eliminating a population at a time or a PC at a time or a combination of both (with replacement) did not significantly change the results (Figs. [Link], [Link], [Link], [Link], [Link], [Link], [Link], [Link], and this held true whether RF was included (Figs. [Link], [Link], [Link], [Link], [Link], [Link], [Link], [Link]) or excluded (Figs. [Link], [Link], [Link], [Link], [Link], [Link], [Link], [Link]). A summary of the results for analyses without RF is given in Table A2.

**Table 2:** Results of Lande's model tests for *Rhinolophus simulator*

Sites	PCs used	Slope *b*	*SE*	*p* (*b ≠ *1)	Correlated PCs	Consistent with drift?
All	All	0.476	0.059	<.05	1–2; 1–9; 3–4; 7–9; 7–10; 8–9; 8–10; 9–10	No
	−11	0.476	0.059	<.05	1–2; 1–9; 3–4; 7–9; 7–10; 8–9; 8–10; 9–10	No
‐CC	All	0.481	0.044	<.05		No
	−11	0.431	0.079	<.05		No
‐DM	All	0.499	0.068	<.05		No
	−11	0.452	0.118	<.05		No
‐KL	All	0.474	0.047	<.05		No
	−11	0.450	0.088	<.05		No
‐LOB	All	0.488	0.064	<.05		No
	−11	0.397	0.130	<.05		No
‐MC	All	0.462	0.067	<.05		No
	−11	0.446	0.128	<.05		No
‐MM	All	0.410	0.079	<.05		No
	−11	0.370	0.149	<.05		No
‐MT	All	0.488	0.052	<.05		No
	−11	0.553	0.097	<.05		No
‐SH	All	0.472	0.063	<.05		No
	−11	0.442	0.115	<.05		No
‐SUD	All	0.441	0.073	<.05		No
	−11	0.357	0.124	<.05		No

NB: Localities: PA = Pafuri, JET = Jiri Estate – Triangle, MM = Monaci Mine, OD = Odzi German Shafts, DM = Dambanzara, MC = Mabura, KP = Kapamukombe, KL = Kalenda, SUD = Sudwala. Starting with all populations/sites (All), and excluding one at a time (e.g., ‐MM means population MM is excluded). The regression was run with either all PCs (PCs used; All) or excluding some PCs (e.g., −11 means exclude PC 11). Slope *b* is the estimation of the regression slope, along with its standard error (*SE*) and *p* (*b *≠* 1)* is the *p* value for the null hypothesis of *b* = 1. Principal components that are significantly correlated at the level of *p* < 0.001 are listed in the column “Correlated PCs”.

**Table 3:** Results of Lande's model tests for *Rhinolophus swinnyi*

Sites	PCs used	Slope *b*	*SE*	*p* (*b ≠ *1)	Correlated PCs	Consistent with drift?
All	All	0.344	0.134	<.05	2–4; 2–7; 5–6; 5–9; 7–11; 9–10	No
	−11	0.344	0.134	<.05	2–4; 2–7; 5–6; 5–9; 9–10	No
‐CC	All	0.330	0.134	<.05		No
	−11	0.116	0.152	<.05		No
‐DM	All	0.329	0.139	<.05		No
	−11	0.107	0.156	<.05		No
‐JET	All	0.190	0.129	<.05		No
	−11	−0.011	0.129	<.05		No
‐KL	All	0.452	0.149	<.05		No
	−11	0.136	0.161	<.05		No
‐KP	All	0.429	0.106	<.05		No
	−11	0.333	0.137	<.05		No
‐MC	All	0.315	0.148	<.05		No
	−11	0.103	0.178	<.05		No
‐OD	All	0.371	0.123	<.05		No
	−11	0.169	0.127	<.05		No
‐PA	All	0.295	0.124	<.05		No
	−11	0.078	0.125	<.05		No

Abbreviations same as in Table 1.

Generally, the last PCs showed a notable disparity between *B* and *W* for very minor eigenvalues and seemed to influence the regression line in both species. Removing the last PCs with high influence but minimal variation explained, in each analysis (Figures 2, 3, 4, 5; Tables 2 and 3), still did not identify any case where drift was supported. Combining this procedure with dropping a population/locality from the model (Figs. [Link], [Link] and [Link], [Link]; Tables 2 and 3) did not change the result.

PC scores for both species (Tables 4; A3 and A4) showed that the maneuverability PCs contributed most of the variance in our data, followed by size and then echolocation behavior; we compiled a summary of this in Table 4, using information from Tables A3 and A4. Maneuverability PCs still ranked higher than size even when RF was excluded from the analysis (Table 4). Additionally, there was an indication of coselection between some PC pairs (Tables 2 and 3), and correlated pairs were highly variable across the different cases analyzed.

**Table 4:**
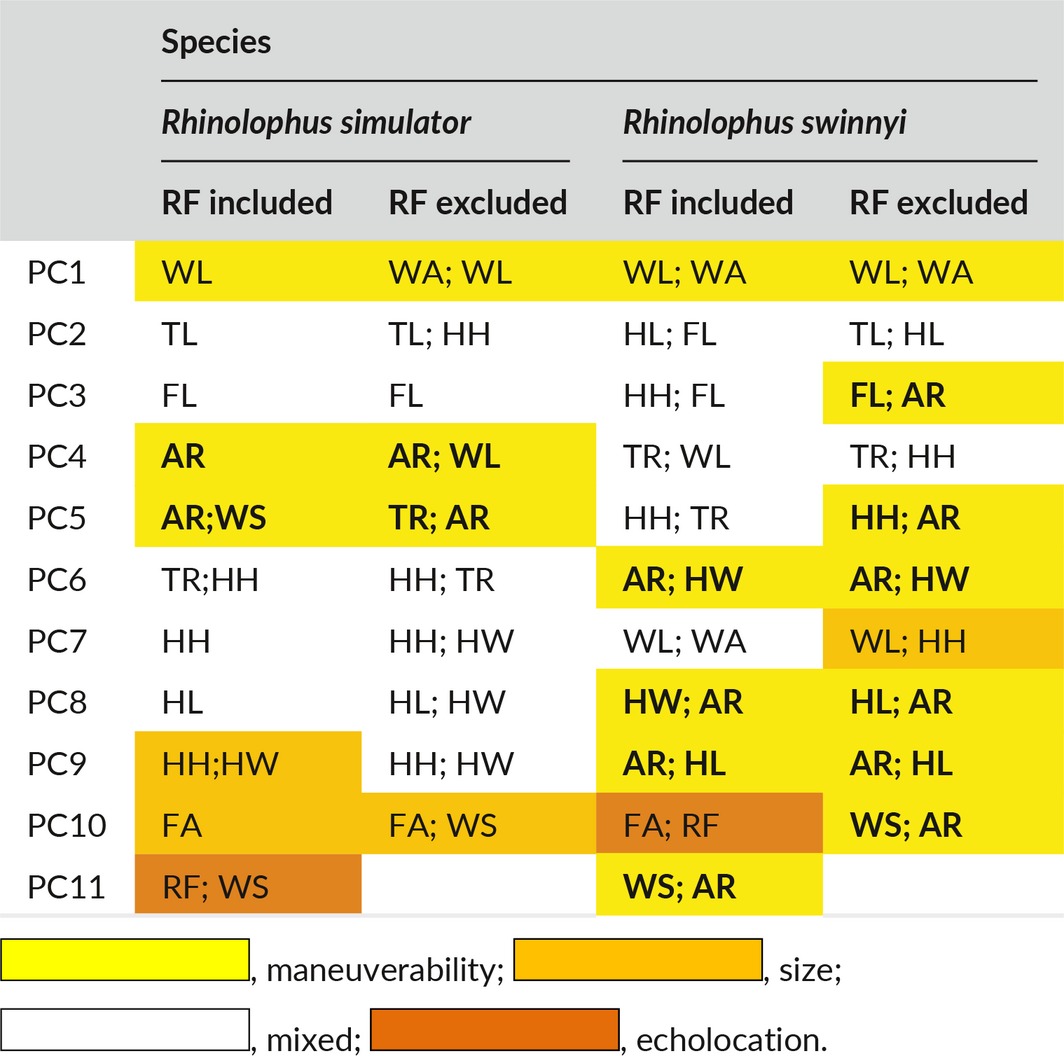
Variables predominantly making up each of the PCs used in the analysis and how these can be related to the bat's functional behavior (color coded, and key provided below the table)

Comparing the results for geographic variation (Figure 1) with those from Lande's Models indicated that the most distinct site for *R. simulator* (MM; Figure 1a) and contrarily, one of the least distinct sites for *R. swinnyi* (PA; Figure 1b) did not significantly influence the relationship between *B* and *W* of the Lande's model results as would have been expected. When these (together with PCs carrying low eigenvalues) were excluded from the model, the results still showed evidence for selection. The geographic variation in phenotype was mainly through maneuverability and echolocation behavior in *R. simulator*, whereas in *R. swinnyi,* it was mainly through echolocation behavior and size (see Figure 1 and the subsection on geographic variation). When RF was excluded from the analyses, there was support for selection, mainly on maneuverability and size, in both species (Figs. [Link], [Link], [Link], [Link], [Link], [Link], [Link], [Link]). Lack of support for drift was also confirmed by evidence for coselection in the form of correlations between PCs. If drift was responsible, these PCs would not be correlated (Ackerman & Cheverud, 2002). Thus, geographic variation and the evidence of coselection among different traits suggest that variation in these two species was predominantly the result of selection.

## 4 Discussion

Selection was the predominant process implicated in phenotypic divergence in *R. simulator* and *R. swinnyi* in accordance with our prediction (1). This result was supported by the absence of a correlation between geographic distance and phenotypic differences across populations for both species. Furthermore, there was strong evidence for coselection among the different traits analyzed supporting no role for drift. Population divergence in both species was the result of habitat mediated selection mostly on flight and maneuverability followed by resting frequency (both species). Contrary to prediction (2), selection was not differentially exerted across populations because there was no significant change in the results when localities were excluded one at a time. Contrary to prediction (3) there was no significant difference (Figures 2, 3, 4, 5) in the relative roles of drift and selection between the two species.

Our results contrast with those from studies on monkeys and humans which report a predominance of drift (Ackermann & Cheverud, 2002, 2004; Smith, 2011). However, evidence for significant deviations from neutrality were also found in craniofacial variation of early and late Holocene Native American groups (de Azevedo et al., 2015). Contrary to our study, these studies also found that selection (and drift) was differentially expressed across different features of the phenotype and/or across different localities (de Azevedo et al., 2015). There are currently no examples of studies in which this approach was used on bats. However, Porto et al. (2015) and Assis, Rossoni, Patton, and Marroig (2016) echo the leading role of selection in marsupial and chipmunk evolution, respectively; using an even more robust approach which incorporated genetics into Lande's based modelling. Differences in the results of our and these studies may also be partly due to their comparison of species, whereas our study compared populations within species. Nonetheless, our results support these and other findings from previous purely genetic approaches on the horseshoe bat, *Rhinolophus ferrumequinum* (Sun et al., 2013), and two species of grasshopper, *Melanoplus sanguinipes* and *M. devastator* (Roff & Mousseau, 2005), which all suggested a predominance of natural selection.

The bat phenotype is characterized by traits that have direct fitness benefits, and it is therefore not surprising that selection rather than drift appears to be the predominant process in the evolution of the bats. Traits associated with flight, feeding, and sensory systems have severe consequences on survival and reproduction both separately and in combination and several adaptive complexes have evolved in bats (Norberg & Rayner, 1987). For example, there are strong correlations between body size and echolocation (Jones, 1996), wing loading and echolocation (Norberg & Rayner, 1987) and skull features associated with feeding and echolocation (Jacobs et al., 2014). The bat phenotype is also characterized by tight associations with environmental factors. There are correlations between habitat and each of wing loading (Kalcounis & Brigham, 1995) and echolocation (Schnitzler & Kalko, 2001) and between echolocation and climatic factors (Mutumi et al., 2016). Bat morphology also correlates with climate following eco‐geographic rules, including Allen's Rule (Solick & Barclay, 2006) and Bergmann's Rule (Hand & York, 1990). In both these studies, phenotypic variation was the result of adaptations for reduced heat loss, for example, *Myotis evotis* had larger ears and wings in mountain populations where it was cooler and wetter than in the lower lying areas. The predominance of selection over drift we report here was also found in at least two other studies that investigated the relative roles of these two processes, Sun et al. (2013) and Odendaal et al. (2014). Both studies concluded that divergent ecological selection rather than drift was responsible for the variation in RF across populations. Although the focus of both these studies and ours was different rhinolophid bat species, there is no reason to suspect that similar results would not be obtained for other bats or any other organism whose life history is dependent on a tight association between phenotypic traits and physical laws. This would especially include those animals that rely on flight, swimming, or have specialized sensory and mating systems (e.g., birds, frogs, fish, and insects).

It is possible that drift may have occurred in parts of the phenotype we did not consider here (e.g., the skull). Drift was detected in the basi‐cranium, temporal bone, and face of modern human populations, and in some features of the skull within primates (Ackermann & Cheverud, 2004; de Azevedo et al., 2015). Even though morphological/phenotypic integration theories specify that a phenotype mostly evolves as whole, other features may still evolve somewhat independently. For example, in mandibles and crania of *Rhinolophus ferumequinum*, two separate modules were identified (Jojic, Budinski, Blagojevic, & Vujosevic, 2015). In the two species we studied, it is evident that both morphology and echolocation are under selection because analyses with and without RF showed similar results. Nevertheless, neutral evolutionary processes may facilitate convergence in morphology among different populations of bats sharing similar ecological contexts but occupying different geographic locations (Jacobs et al., 2013). There is therefore need for a partitioned analysis to investigate different structures of the phenotype separately. Models can be structured to analyze traits associated with different functional complexes within the skull, flight apparatus, and perhaps also within echolocation call features. Such an approach is not possible with the data at hand and would require more advanced equipment to maybe perform 3D scans of live bats in the field. This would provide higher resolution and more data points (of high accuracy) from functional complexes. However, the results presented here still provide a valuable overview for analyzing microevolutionary signatures responsible for phenotypic diversification.

The signal for selection was not the same across traits. Selection was greatest on maneuverability and size than on RF in both species highlighting the significant role that flight plays in the survival of bats (Norberg & Rayner, 1987) and to some extent, the importance of sensory drive in the diversification of organisms (Mutumi et al., 2016). The RF of the echolocation calls of both species is influenced by climate mediated selection (Mutumi et al., 2016). *Rhinolophus swinnyi* used lower frequency calls in cooler, humid areas than in hot dry areas, whereas *R. simulator* showed spatial structuring by latitude (Mutumi et al., 2016). Even though other stochastic factors may be responsible for the divergence in the phenotype of these two species, results in the current study indicate that sensory‐based selection drives the divergence and that echolocation and flight behavior play a pivotal role.

Despite differences in call frequency, selection was not more pronounced on the RF of *R. swinnyi* than on *R. simulator* (Figures 2, 3, 4, 5; Tables 2 and 3). *R. swinnyi* uses higher RF than *R. simulator* meaning its echolocation experiences increased atmospheric attenuation and it would be expected that the RF of *R. swinnyi* would be under more stringent selection. However, the difference in echolocation between *R. swinnyi* and *R. simulator* (20 kHz) translates to only 1.16 mm difference in wavelength (http://www.wavelengthcalculator.com) and may not be large enough to equate to significant differences in their sensory or foraging ecology, for example, differences in prey sizes or habitat (Jacobs et al., 2007). Future research should compare species that have a substantial difference in the frequencies of their RF at lower ranges of the frequency spectrum, that is, ≤80 kHz (Jacobs et al., 2007). Such comparisons would involve differences in wavelengths which may be ecologically significant.

In contrast to that on RF, selection on traits associated with maneuverability and size differed between the two species. Selection was more pronounced on maneuverability than body size in *R. simulator* but the reverse was true for *R. swinnyi*. An explanation for these differences requires more detailed analyses of their habitats and, more importantly, of how these two species use their habitats.

The strong signal for selection suggests that the populations may be isolated enough so that the counteracting effects of gene flow are relatively low compared to the effects of selection pressure experienced by these populations. It is therefore likely that the phenotypic divergence reported in this study is a result of adaptation to local habitats reinforced by limited gene flow among populations which allows adaptive differences to accumulate. The vicariance responsible for the reduced gene flow cannot be a result of isolation by distance because the mantel test results did not show a correlation between geographic distance and phenotypic distance. The absence of such a correlation suggests that there are other barriers to dispersal. Localities closest to each other (fig. 1 in Mutumi et al., 2016) were not necessarily the most similar, for example, CC–MC for *R. simulator* and KP–DM for *R. swinnyi* (Fig 1). The topography between these sites showed that each pair is separated by an extensive mountain range. For example, in CC–MC pair, MC is in a low valley (the Sanyati cotton belt, Zimbabwe) and CC is situated in the northern part of the watershed of Zimbabwe which has the highest elevation in the country. Similarly, DM is separated from KP by the Matusadonha mountain range, Zimbabwe.

Relative to the rest of the other localities, *R. swinnyi* from KP have the larger measurements for 50% of the parameters measured (FA, HH, HL, TL, WS, WL; Table A1) and have the second lowest echolocation frequency, 103.28 kHz. This may be related to the situation of KP in an ecotone of two ecoregions (Zambezian/Mopane and Southern Miombo woodlands). Ecotones characteristically present diverse selective forces which may act as ecological barriers to gene flow (Harris & Reed, 2002). Similarly, LOB sits in an ecotone of three vegetation biomes (Kalahari Acacia‐Baikiaea woodlands, Kalahari xeric Savannah, and the Southern Africa bushveld). Such ecological barriers to gene flow between LOB and the other nearby localities may make them more divergent. LOB has the highest RF and GKC the second highest, whereas MM has the lowest FA, HH, HL, and the highest FL and AR making these three sites different from the other populations of *R. simulator*. These differences are partly explained by differences in climatic variables but competition for discrete frequency bands in a social context (Bastian & Jacobs, 2015; Mutumi et al., 2016), or isolation by habitat/ecology (Wang, 2013; Wang, Glor, & Losos, 2013) may contribute to these differences. However, a more detailed analysis of the environment (including a consideration of co‐existing congenerics) and the manner in which these bats use the environment need to be undertaken before these differences can be explained.

## 5 Limitations

Support for selection on morphological traits when RF was excluded from the analyses may be due to the low number of morphological variables available to us because of the practicalities of collecting data from live bats under difficult field conditions without compromising the welfare of the bats. A rigorous determination of which functional complex of the phenotype selection acts upon, and/or the relative influence of selection and drift on each of these, requires several variables for each functional complex. Such data are not currently available for our focal species even from museum collections.

Furthermore, our analyses could have been impacted by small sample sizes encountered at some sites which may have incorrectly determined the phenotypic means. Similarly, the low number of variables may underestimate the regression between B and W and outliers could have exerted undue influence on the slopes of these regressions. However, our analysis addressed the effect of outliers and low variable numbers through the sensitivity test (analysis by exclusion of a site or a PC at a time). These analyses did not change the results significantly showing that our variable numbers and perhaps even sample sizes were adequate. Overall the main focus of our study was to apply a rigorous method for detecting natural selection in quantitative traits of horseshoe bats, and for this purpose, our data and the methods appeared to be adequate.

## 6 Conclusion

In organisms with phenotypes that are highly sensitive to selection owing to the combined use of sophisticated sensory and locomotor systems (e.g., insects, frogs, birds, and bats), selection rather than drift is still likely to be the predominant process in the evolution of phenotypic variation and ultimately lineage divergence, perhaps even when population sizes are small. Drift is therefore only likely to exert an influence on traits that do not have a severe impact on fitness.

## Conflict of interest

None declared.

## Authors’ contributions

GLM and DSJ conceived and designed the study and carried out the fieldwork. GLM and HW conducted the ecological analyses with input from DSJ. GLM, DSJ, and HW contributed to the writing of the manuscript and read and approved the final manuscript.

## Ethical statement

Capture, handling, and voucher collection methods of this research complied with the guidelines recommended by the American Society of Mammalogists (Gannon and Sikes, 2007), and sampling guidelines compiled by Aegerter and Heritage (2005) and Kunz and Parsons (2009); and were approved by the Science Faculty Animal Ethics Committee at the University of Cape Town (Clearance Number 2013/2011/V6/DJ). All workers handling bats were vaccinated for rabies and were required to use protective gloves when handling bats and samples.
