# Supplementary material for: The relative contribution of drift and selection to phenotypic divergence: A test case using the horseshoe bats Rhinolophus simulator and Rhinolophus swinnyi
Source: Ecol Evol. 2017 May 9;7(12):4299–311. doi: 10.1002/ece3.2966 (PMC5478076; doi:10.1002/ece3.2966)
Supplement: Supplementary file 11 [file ECE3-7-4299-s003.docx]

**Table A1:** Means and standard deviations (±SD) of phenotypic parameters measured from live bats in the field.

| ***Rhinolophus simulator*** | | | | | | | | | | | | | | | | | | | | | | | | | |
| --- | --- | --- | --- | --- | --- | --- | --- | --- | --- | --- | --- | --- | --- | --- | --- | --- | --- | --- | --- | --- | --- | --- | --- | --- | --- |
| **StudySite** | **N** | **RF** | **±SD** | **FA** | **±SD** | **TR** | **±SD** | **HH** | **±SD** | **HW** | **±SD** | **HL** | **±SD** | **FL** | **±SD** | **TL** | **±SD** | **WS** | **±SD** | **WA** | **±SD** | **AR** | **±SD** | **WL** | **±SD** |
| **CC** | 8 | 80.14 | 0.59 | 44.29 | 1.07 | 6.58 | 0.54 | 7.38 | 0.84 | 9.01 | 0.57 | 18.37 | 2.45 | 7.40 | 0.94 | 21.93 | 3.11 | 0.26 | 0.0123 | 0.0134 | 0.0011 | 5.09 | 0.24 | 5.00 | 0.40 |
| **DM** | 22 | 79.18 | 0.95 | 44.45 | 1.21 | 6.75 | 0.39 | 7.00 | 0.39 | 9.04 | 0.60 | 19.57 | 1.17 | 7.55 | 0.81 | 23.13 | 2.73 | 0.26 | 0.0115 | 0.0133 | 0.0010 | 4.94 | 0.35 | 5.02 | 0.42 |
| **KL** | 6 | 77.78 | 0.54 | 45.12 | 0.97 | 6.96 | 0.52 | 6.70 | 0.24 | 9.50 | 0.52 | 18.93 | 1.08 | 7.97 | 0.74 | 26.87 | 1.71 | 0.25 | 0.0230 | 0.0132 | 0.0019 | 4.91 | 0.36 | 6.77 | 1.47 |
| **LOB** | 18 | 84.61 | 0.64 | 45.03 | 1.04 | 7.13 | 0.57 | 6.98 | 0.49 | 8.88 | 0.62 | 17.89 | 0.88 | 8.25 | 0.84 | 24.38 | 2.00 | 0.28 | 0.0110 | 0.0138 | 0.0011 | 5.74 | 0.47 | 5.53 | 0.99 |
| **MC** | 8 | 79.53 | 0.77 | 45.28 | 1.27 | 6.51 | 0.45 | 7.30 | 0.31 | 8.96 | 0.29 | 20.10 | 0.37 | 7.41 | 0.76 | 20.78 | 2.00 | 0.26 | 0.0211 | 0.0132 | 0.0019 | 5.15 | 0.49 | 4.99 | 0.75 |
| **MM** | 4 | 81.25 | 0.66 | 44.00 | 1.15 | 7.08 | 0.47 | 6.55 | 0.25 | 9.28 | 0.26 | 16.50 | 1.32 | 9.93 | 0.51 | 25.90 | 1.98 | 0.27 | 0.0058 | 0.0120 | 0.0003 | 6.00 | 0.36 | 5.31 | 0.49 |
| **MT** | 10 | 78.32 | 0.76 | 46.64 | 1.21 | 7.19 | 0.45 | 7.70 | 0.86 | 9.60 | 0.69 | 19.55 | 1.10 | 8.35 | 1.11 | 31.19 | 4.51 | 0.26 | 0.0181 | 0.0127 | 0.0011 | 5.16 | 0.61 | 7.26 | 0.76 |
| **SH** | 9 | 78.79 | 0.51 | 44.14 | 1.49 | 6.58 | 0.64 | 6.97 | 0.43 | 9.27 | 0.56 | 18.66 | 1.41 | 7.66 | 0.75 | 25.85 | 1.68 | 0.27 | 0.0116 | 0.0141 | 0.0010 | 5.14 | 0.21 | 5.65 | 0.45 |
| **SUD** | 16 | 80.74 | 0.81 | 44.47 | 1.31 | 6.81 | 0.59 | 7.03 | 0.51 | 9.44 | 0.36 | 18.33 | 1.11 | 7.80 | 0.58 | 25.99 | 1.64 | 0.27 | 0.0103 | 0.0141 | 0.0009 | 4.98 | 0.37 | 4.72 | 0.23 |
| **Total N** | 101 |  |  |  |  |  |  |  |  |  |  |  |  |  |  |  |  |  |  |  |  |  |  |  |  |
| ***Rhinolophus swinnyi*** | | | | | | | | | | | | | | | | | | | | | | | | | |
| **CC** | 4 | 106.55 | 0.91 | 41.43 | 1.34 | 6.68 | 0.62 | 7.23 | 1.14 | 9.05 | 0.29 | 19.10 | 1.19 | 7.33 | 1.11 | 20.60 | 2.84 | 0.25 | 0.0137 | 0.0120 | 0.0005 | 5.07 | 0.37 | 4.76 | 0.55 |
| **DM** | 4 | 104.23 | 1.99 | 42.13 | 0.76 | 5.88 | 0.52 | 6.65 | 0.44 | 8.50 | 0.36 | 17.90 | 1.22 | 7.08 | 0.81 | 23.23 | 3.07 | 0.24 | 0.0073 | 0.0114 | 0.0005 | 5.13 | 0.25 | 4.70 | 0.28 |
| **JET** | 20 | 103.97 | 1.98 | 42.17 | 1.81 | 6.47 | 0.56 | 7.10 | 0.66 | 8.60 | 0.44 | 19.92 | 1.17 | 7.19 | 0.81 | 23.72 | 2.55 | 0.25 | 0.0129 | 0.0122 | 0.0012 | 5.13 | 0.34 | 4.95 | 0.55 |
| **KL** | 8 | 102.83 | 0.77 | 42.91 | 1.18 | 6.21 | 0.31 | 6.61 | 0.33 | 8.63 | 0.32 | 16.63 | 0.78 | 7.84 | 0.76 | 24.29 | 1.48 | 0.25 | 0.0099 | 0.0124 | 0.0013 | 5.31 | 0.22 | 5.37 | 0.52 |
| **KP** | 14 | 103.28 | 1.55 | 45.49 | 1.36 | 6.54 | 0.46 | 7.39 | 0.51 | 8.96 | 0.33 | 20.67 | 1.03 | 7.34 | 0.52 | 24.91 | 1.14 | 0.27 | 0.0067 | 0.0131 | 0.0011 | 5.51 | 0.33 | 5.59 | 0.49 |
| **MC** | 9 | 104.98 | 1.14 | 41.70 | 0.50 | 6.30 | 0.53 | 6.89 | 0.20 | 9.02 | 0.17 | 18.59 | 1.44 | 7.46 | 0.75 | 21.18 | 2.42 | 0.24 | 0.0054 | 0.0111 | 0.0011 | 5.38 | 0.42 | 5.13 | 0.78 |
| **OD** | 33 | 103.50 | 1.26 | 41.25 | 0.97 | 6.49 | 0.59 | 6.85 | 0.45 | 8.33 | 0.74 | 18.94 | 1.37 | 7.43 | 0.66 | 20.54 | 2.69 | 0.25 | 0.0107 | 0.0114 | 0.0010 | 5.56 | 0.37 | 4.73 | 0.45 |
| **PA** | 33 | 104.31 | 1.52 | 40.94 | 0.74 | 6.23 | 0.50 | 6.52 | 0.34 | 8.58 | 0.31 | 18.19 | 1.20 | 7.26 | 0.73 | 22.04 | 2.05 | 0.25 | 0.0110 | 0.0123 | 0.0007 | 5.16 | 0.45 | 4.24 | 0.35 |
| **Total N** | 125 |  |  |  |  |  |  |  |  |  |  |  |  |  |  |  |  |  |  |  |  |  |  |  |  |

Abbreviations for phenotypic parameters are given in table A1. Study site abreviations: PA = Pafuri, JET = Jiri Estate – Triangle, MM = Monaci Mine, OD = Odzi German Shafts, DM = Dambanzara, MC = Mabura, KP = Kapatamukombe, KL = Kalenda, SUD = Sudwala. RF was measured in kilohertz (kHz), FA, TR, HH, HW, HL, FL and TL in millimetres (mm), WS in meters, Wing Area in square meters, Aspect Ratio as wingspan squared divided by wing area, WL was expressed in Newtons per square meter.
