# Supplementary material for: The relative contribution of drift and selection to phenotypic divergence: A test case using the horseshoe bats Rhinolophus simulator and Rhinolophus swinnyi
Source: Ecol Evol. 2017 May 9;7(12):4299–311. doi: 10.1002/ece3.2966 (PMC5478076; doi:10.1002/ece3.2966)
Supplement: Supplementary file 12 [file ECE3-7-4299-s012.docx]

**Table A2a:** Results of Lande’s model tests for *Rhinolophus simulator* without RF in the analysis

| Sites | PCs Used | Slope *b* | S.E. | *p*(*b ≠*1 | Correlated PCs | Consistent with drift? |
| --- | --- | --- | --- | --- | --- | --- |
| All | All | 0.210 | 0.079 | < 0.05 | 1-2; 1-9; 1-10; 2-9; 3-4; 7-10; 8-9; 8-10; 9-10. | No |
|  | -10 | 0.210 | 0.079 | < 0.05 | 1-2; 1-9; 2-9; 3-4; 8-9 | No |
| -CC | All | 0.206 | 0.062 | < 0.05 |  | No |
|  | -10 | 0.372 | 0.106 | < 0.05 |  | No |
| -DM | All | 0.234 | 0.083 | < 0.05 |  | No |
|  | -10 | 0.299 | 0.166 | < 0.05 |  | No |
| -KL | All | 0.214 | 0.069 | < 0.05 |  | No |
|  | -10 | 0.332 | 0.147 | < 0.05 |  | No |
| -LOB | All | 0.126 | 0.088 | < 0.05 |  | No |
|  | -10 | 0.301 | 0.199 | < 0.05 |  | No |
| -MC | All | 0.208 | 0.087 | < 0.05 |  | No |
|  | -10 | 0.346 | 0.189 | < 0.05 |  | No |
| -MM | All | 0.165 | 0.110 | < 0.05 |  | No |
|  | -10 | 0.307 | 0.230 | < 0.05 |  | No |
| -MT | All | 0.395 | 0.068 | < 0.05 |  | No |
|  | -10 | 0.404 | 0.175 | < 0.05 |  | No |
| -SH | All | 0.223 | 0.087 | < 0.05 |  | No |
|  | -10 | 0.338 | 0.185 | < 0.05 |  | No |
| -SUD | All | 0.175 | 0.090 | < 0.05 |  | No |
|  |  | 0.258 | 0.186 | < 0.05 |  | No |

**NB:** We only showed PCs which when removed changed the relationship between B and W (9 and 11). Localities: PA = Pafuri, JET = Jiri Estate – Triangle, MM = Monaci Mine, OD = Odzi German Shafts, DM = Dambanzara, MC = Mabura, KP = Kapamukombe, KL = Kalenda, SUD = Sudwala. Pop = Populations (N – number of) included, starting with all populations (All), and excluding one at a time (e.g., -MM = meaning Population MM is excluded). The regression is run with either all PCs (PCs used; All) or excluding some PCs (Exclude e.g.., 9 & 11 = -9&11). Slope *b*: estimation of regression slope, along with the standard error S.E.) and *p (b ≠ 1) p*-value for the null hypothesis of *b* = 1. Principal components presenting significant correlations with *p* < 0.001 (Correlated PCs).The models in bold are consistent with the Null model of drift.

**Table A2b:** Results of Lande’s model tests for *Rhinolophus swinnyi* without RF in the analysis*.*

| Sites | PCs Used | Slope *b* | S.E. | *p*(*b ≠*1) | Correlated PCs | Consistent with drift? |
| --- | --- | --- | --- | --- | --- | --- |
| All | All | 0.186 | 0.127 | < 0.05 | 1-2; 1-9; 1-10; 2-9; 3-4; 7-10; 8-9; 8-10; 9-10 | No |
|  | -10 | 0.186 | 0.127 | < 0.05 | 1-2; 1-9; 2-9; 3-4; 8-9 | No |
| -CC | All | 0.152 | 0.125 | < 0.05 |  | No |
|  | -10 | -0.034 | 0.189 | < 0.05 |  | No |
| -DM | All | 0.176 | 0.130 | < 0.05 |  | No |
|  | -10 | -0.009 | 0.199 | < 0.05 |  | No |
| -JET | All | 0.217 | 0.127 | < 0.05 |  | No |
|  | -10 | -0.021 | 0.156 | < 0.05 |  | No |
| -KL | All | 0.139 | 0.120 | < 0.05 |  | No |
|  | -10 | 0.036 | 0.214 | < 0.05 |  | No |
| -KP | All | 0.312 | 0.123 | < 0.05 |  | No |
|  | -10 | 0.123 | 0.180 | < 0.05 |  | No |
| -MC | All | 0.123 | 0.145 | < 0.05 |  | No |
|  | -10 | -0.072 | 0.227 | < 0.05 |  | No |
| -OD | All | 0.301 | 0.122 | < 0.05 |  | No |
|  | -10 | 0.089 | 0.155 | < 0.05 |  | No |
| -PA | All | 0.150 | 0.110 | < 0.05 |  | No |
|  | -10 | -0.077 | 0.141 | < 0.05 |  | No |

Abbreviations same as in table 1
