# Supplementary material for: The relative contribution of drift and selection to phenotypic divergence: A test case using the horseshoe bats Rhinolophus simulator and Rhinolophus swinnyi
Source: Ecol Evol. 2017 May 9;7(12):4299–311. doi: 10.1002/ece3.2966 (PMC5478076; doi:10.1002/ece3.2966)
Supplement: Supplementary file 13 [file ECE3-7-4299-s013.docx]

**Table A3:** Principal component factor scores for *Rhinolophus simulator* with and without RF in the analyses

| ***Rhinolophus simulator, analysis including RF*** | | | | | | | | | | | | ***Rhinolophus simulator, analysis excluding RF*** | | | | | | | | | |
| --- | --- | --- | --- | --- | --- | --- | --- | --- | --- | --- | --- | --- | --- | --- | --- | --- | --- | --- | --- | --- | --- |
| **PC** | **1** | **2** | **3** | **4** | **5** | **6** | **7** | **8** | **9** | **10** | **11** | **1** | **2** | **3** | **4** | **5** | **6** | **7** | **8** | **9** | **10** |
| FA | 0.012 | 0.013 | 0.011 | 0.004 | 0.033 | 0.008 | 0.006 | 0.015 | 0.031 | 0.458 | 0.005 | 0.013 | 0.012 | 0.010 | 0.006 | 0.035 | 0.005 | 0.007 | 0.012 | 0.141 | 0.362 |
| TR | 0.025 | 0.027 | 0.019 | 0.104 | 0.324 | 0.236 | 0.072 | 0.006 | 0.126 | 0.016 | 0.001 | 0.024 | 0.028 | 0.026 | 0.099 | 0.333 | 0.211 | 0.104 | 0.014 | 0.138 | 0.016 |
| HH | 0.082 | 0.164 | 0.092 | 0.108 | 0.014 | 0.304 | 0.420 | 0.040 | 0.244 | 0.035 | 0.004 | 0.084 | 0.172 | 0.086 | 0.110 | 0.019 | 0.388 | 0.372 | 0.094 | 0.221 | 0.078 |
| HL | 0.035 | 0.084 | 0.045 | 0.012 | 0.044 | 0.112 | 0.213 | 0.112 | 0.267 | 0.014 | 0.007 | 0.036 | 0.084 | 0.046 | 0.009 | 0.043 | 0.086 | 0.237 | 0.173 | 0.219 | 0.058 |
| HW | 0.007 | 0.125 | 0.015 | 0.070 | 0.025 | 0.080 | 0.124 | 0.365 | 0.056 | 0.008 | 0.002 | 0.008 | 0.127 | 0.008 | 0.065 | 0.025 | 0.074 | 0.146 | 0.345 | 0.121 | 0.025 |
| FL | 0.044 | 0.027 | 0.338 | 0.035 | 0.004 | 0.060 | 0.001 | 0.020 | 0.027 | 0.011 | 0.002 | 0.043 | 0.021 | 0.343 | 0.027 | 0.004 | 0.063 | 0.011 | 0.029 | 0.010 | 0.011 |
| TL | 0.068 | 0.363 | 0.010 | 0.116 | 0.014 | 0.029 | 0.110 | 0.130 | 0.016 | 0.011 | 0.004 | 0.065 | 0.343 | 0.008 | 0.107 | 0.015 | 0.045 | 0.097 | 0.120 | 0.047 | 0.003 |
| WS | 0.070 | 0.044 | 0.008 | 0.115 | 0.169 | 0.142 | 0.008 | 0.006 | 0.086 | 0.032 | 0.363 | 0.069 | 0.045 | 0.001 | 0.100 | 0.162 | 0.138 | 0.031 | 0.017 | 0.086 | 0.247 |
| WA | 0.174 | 0.034 | 0.008 | 0.130 | 0.143 | 0.156 | 0.075 | 0.055 | 0.161 | 0.005 | 0.146 | 0.157 | 0.032 | 0.011 | 0.134 | 0.119 | 0.118 | 0.084 | 0.022 | 0.152 | 0.037 |
| A | 0.077 | 0.074 | 0.033 | 0.532 | 0.231 | 0.112 | 0.123 | 0.127 | 0.007 | 0.012 | 0.286 | 0.051 | 0.053 | 0.011 | 0.355 | 0.164 | 0.106 | 0.060 | 0.075 | 0.004 | 0.101 |
| WL | 0.433 | 0.086 | 0.089 | 0.110 | 0.130 | 0.111 | 0.002 | 0.075 | 0.137 | 0.023 | 0.002 | 0.369 | 0.075 | 0.075 | 0.106 | 0.103 | 0.085 | 0.008 | 0.040 | 0.121 | 0.042 |
| RF | 0.004 | 0.009 | 0.002 | 0.001 | 0.004 | 0.012 | 0.008 | 0.016 | 0.013 | 0.061 | 0.185 | 0.013 | 0.012 | 0.010 | 0.006 | 0.035 | 0.005 | 0.007 | 0.012 | 0.141 | 0.362 |
