# Supplementary material for: The relative contribution of drift and selection to phenotypic divergence: A test case using the horseshoe bats Rhinolophus simulator and Rhinolophus swinnyi
Source: Ecol Evol. 2017 May 9;7(12):4299–311. doi: 10.1002/ece3.2966 (PMC5478076; doi:10.1002/ece3.2966)
Supplement: Supplementary file 14 [file ECE3-7-4299-s014.docx]

**Table A4:** Principal component factor scores for *Rhinolophus swinnyi* with and without including RF in the analyses

| ***Rhinolophus swinnyi, analysis including RF*** | | | | | | | | | | | | ***Rhinolophus swinnyi, analysis excluding RF*** | | | | | | | | | |
| --- | --- | --- | --- | --- | --- | --- | --- | --- | --- | --- | --- | --- | --- | --- | --- | --- | --- | --- | --- | --- | --- |
| **PC** | **1** | **2** | **3** | **4** | **5** | **6** | **7** | **8** | **9** | **10** | **11** | **1** | **2** | **3** | **4** | **5** | **6** | **7** | **8** | **9** | **10** |
| FA | 0.012 | 0.013 | 0.011 | 0.065 | 0.014 | 0.002 | 0.085 | 0.014 | 0.021 | 0.400 | 0.057 | 0.012 | 0.013 | 0.012 | 0.065 | 0.013 | 0.005 | 0.085 | 0.010 | 0.161 | 0.082 |
| TR | 0.053 | 0.020 | 0.066 | 0.334 | 0.214 | 0.110 | 0.057 | 0.095 | 0.035 | 0.037 | 0.012 | 0.053 | 0.025 | 0.063 | 0.340 | 0.206 | 0.108 | 0.056 | 0.097 | 0.036 | 0.007 |
| HH | 0.004 | 0.023 | 0.110 | 0.160 | 0.296 | 0.142 | 0.164 | 0.064 | 0.040 | 0.001 | 0.017 | 0.004 | 0.030 | 0.104 | 0.159 | 0.305 | 0.139 | 0.167 | 0.057 | 0.046 | 0.011 |
| HL | 0.028 | 0.027 | 0.072 | 0.074 | 0.056 | 0.184 | 0.116 | 0.308 | 0.005 | 0.002 | 0.027 | 0.028 | 0.027 | 0.076 | 0.073 | 0.065 | 0.175 | 0.123 | 0.312 | 0.039 | 0.020 |
| HW | 0.044 | 0.141 | 0.036 | 0.007 | 0.019 | 0.146 | 0.157 | 0.147 | 0.243 | 0.054 | 0.000 | 0.044 | 0.143 | 0.044 | 0.005 | 0.027 | 0.142 | 0.157 | 0.176 | 0.195 | 0.040 |
| FL | 0.015 | 0.107 | 0.287 | 0.040 | 0.052 | 0.133 | 0.016 | 0.048 | 0.018 | 0.005 | 0.002 | 0.015 | 0.098 | 0.293 | 0.044 | 0.049 | 0.139 | 0.014 | 0.040 | 0.015 | 0.003 |
| TL | 0.112 | 0.274 | 0.095 | 0.050 | 0.037 | 0.015 | 0.067 | 0.056 | 0.098 | 0.013 | 0.001 | 0.113 | 0.281 | 0.087 | 0.051 | 0.037 | 0.015 | 0.067 | 0.068 | 0.083 | 0.012 |
| WS | 0.056 | 0.016 | 0.050 | 0.018 | 0.037 | 0.056 | 0.132 | 0.049 | 0.169 | 0.070 | 0.324 | 0.058 | 0.020 | 0.050 | 0.019 | 0.041 | 0.067 | 0.134 | 0.032 | 0.182 | 0.322 |
| WA | 0.222 | 0.066 | 0.006 | 0.081 | 0.072 | 0.065 | 0.192 | 0.027 | 0.114 | 0.028 | 0.175 | 0.200 | 0.062 | 0.000 | 0.070 | 0.061 | 0.055 | 0.172 | 0.020 | 0.117 | 0.150 |
| A | 0.146 | 0.047 | 0.130 | 0.075 | 0.186 | 0.252 | 0.005 | 0.191 | 0.263 | 0.057 | 0.209 | 0.129 | 0.037 | 0.116 | 0.063 | 0.166 | 0.239 | 0.003 | 0.132 | 0.209 | 0.212 |
| WL | 0.333 | 0.149 | 0.083 | 0.172 | 0.117 | 0.121 | 0.200 | 0.018 | 0.079 | 0.058 | 0.029 | 0.311 | 0.139 | 0.078 | 0.157 | 0.108 | 0.113 | 0.188 | 0.014 | 0.096 | 0.015 |
| RF | 0.010 | 0.015 | 0.002 | 0.013 | 0.026 | 0.028 | 0.022 | 0.007 | 0.019 | 0.106 | 0.030 | 0.012 | 0.013 | 0.012 | 0.065 | 0.013 | 0.005 | 0.085 | 0.010 | 0.161 | 0.082 |
